# Poly[[(μ-3,4-dicarboxy­tetra­hydro­furan-2,5-dicarboxyl­ato-κ^4^ *O* ^1^,*O* ^2^,*O* ^5^:*O* ^2′^)(1,10-phenanthroline-κ^2^ *N*,*N*′)copper(II)] 0.69-hydrate]

**DOI:** 10.1107/S1600536808027918

**Published:** 2008-09-06

**Authors:** Yuanqi Lü

**Affiliations:** a Department of Chemistry, Dezhou University, University West Road 566, Dezhou, 253023, People’s Republic of China

## Abstract

In the crystal structure of the title compound, {[Cu(C_8_H_6_O_9_)(C_12_H_8_N_2_)]·0.69H_2_O}_*n*_, the Cu^II^ atom has a distorted octa­hedral geometry, coordinated by four O atoms from two 3,4-dicarboxy­tetra­hydro­furan-2,5-dicarboxyl­ate ligands and two N atoms from one 1,10-phenanthroline ligand. One of the carboxylate groups bridges the Cu^II^ atoms, forming a zigzag chain running along the *b* axis. The chains are linked by a π–π inter­action between aromatic rings with a centroid-to-centroid distance of 3.567 (1) Å, and by hydrogen bonds between the carboxyl­ate group and the disordered water mol­ecule, forming a three-dimensional network.

## Related literature

For related literature, see: Guillem *et al.* (1993 ▶).
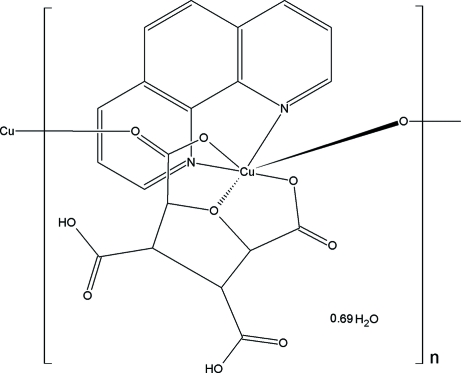


## Experimental

### 

#### Crystal data

[Cu(C_8_H_6_O_9_)(C_12_H_8_N_2_)]·0.69H_2_O
*M* *_r_* = 502.30
Monoclinic, 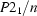

*a* = 12.9215 (7) Å
*b* = 8.5454 (5) Å
*c* = 17.597 (1) Å
β = 90.960 (3)°
*V* = 1942.85 (19) Å^3^
*Z* = 4
Mo *K*α radiation
μ = 1.19 mm^−1^
*T* = 296 (2) K
0.20 × 0.16 × 0.08 mm

#### Data collection

Bruker SMART CCD area-detector diffractometer
Absorption correction: multi-scan (*SADABS*; Sheldrick, 1996 ▶) *T*_min_ = 0.797, *T*_max_ = 0.910
24533 measured reflections
4444 independent reflections
3829 reflections with *I* > 2σ(*I*)
*R*_int_ = 0.020

#### Refinement

*R*[*F* ^2^ > 2σ(*F* ^2^)] = 0.034
*wR*(*F* ^2^) = 0.104
*S* = 1.06
4444 reflections
306 parameters
3 restraints
H atoms treated by a mixture of independent and constrained refinement
Δρ_max_ = 0.91 e Å^−3^
Δρ_min_ = −0.39 e Å^−3^

### 

Data collection: *SMART* (Bruker, 2007 ▶); cell refinement: *SAINT-Plus* (Bruker, 2007 ▶); data reduction: *SAINT-Plus*; program(s) used to solve structure: *SHELXS97* (Sheldrick, 2008 ▶); program(s) used to refine structure: *SHELXL97* (Sheldrick, 2008 ▶); molecular graphics: *SHELXTL* (Sheldrick, 2008 ▶); software used to prepare material for publication: *SHELXTL*.

## Supplementary Material

Crystal structure: contains datablocks global, I. DOI: 10.1107/S1600536808027918/is2325sup1.cif

Structure factors: contains datablocks I. DOI: 10.1107/S1600536808027918/is2325Isup2.hkl

Additional supplementary materials:  crystallographic information; 3D view; checkCIF report

## Figures and Tables

**Table 1:** Hydrogen-bond geometry (Å, °)

*D*—H⋯*A*	*D*—H	H⋯*A*	*D*⋯*A*	*D*—H⋯*A*
O10—H10*A*⋯O5^i^	0.89 (2)	2.47 (10)	2.793 (6)	102 (7)
O6—H6⋯O2^ii^	0.82	1.84	2.646 (2)	166
O3—H3⋯O2	0.82	1.83	2.641 (2)	172

Symmetry codes: (i) 
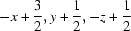
; (ii) 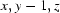.

**Fig. 1.**
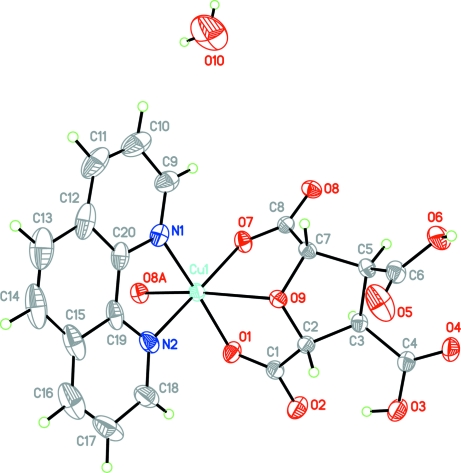
The molecular structure of the title compound, showing 50% probability displacement ellipsoids. The unlabeled atoms are derived from the reference atoms by means of the (3/2 - x, 1/2 + y, 1/2 - z) symmetry transformation.

**Fig. 2.**
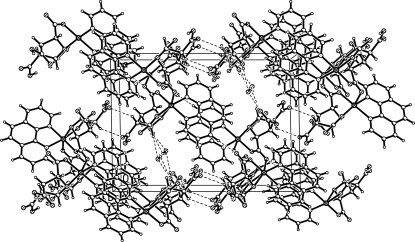
A packing diagram, viewed down the b axis, The hydrogen bonds are shown as dotted lines.

## supplementary crystallographic information

### Comment

The title compound is an infinite zigzag chain structure. The Cu(II) atom in the structure is coordinated by four O atoms form two different deprotonated tetrahydrofuran-2,3,4,5-tetracarboxylic acid and two N atoms from a 1,10-phenanthroline molecule. Two N atoms and two carboxyl O atoms occupy the equatorial plane, while the axial positions are occupied by the furan O atom and the carboxyl O atom from another tetrahydrofuran-2,3,4,5-tetracarboxylate ligand. The bond distances are comparable to the structures reported by Guillem *et al.* (1993). The Cu atom has an octahedral coordination with the pronounced tetragonal distortion (Fig. 1).

The tetrahydrofuran-2,3,4,5-tetracarboxylate ligand coordinated to two Cu(II) atoms at the same time. While one deprotonated carboylate group coordinated to Cu(II) in a monodentate mode, the other one links two adjacent Cu(II) atoms in a bridging mode into an infinite zigzag chain along the *b* axis. Strong π–π interactions between the adjacent chains are indicated by the short distance value of 3.501 (3) Å between the adjacent 1,10-phenanthroline molecules from different chains. The distance between two centres of the overlapped phenyl rings equals to 3.567 (1) Å. Chains are packed together by the π–π interactions into sheets parallel to the (101) plane. Strong hydrogen bonds between H_2_O and carboxylate groups and between different carboxylate groups are observed and the sheets along (101) are linked together by the hydrogen bonds into a three-dimensional framework (Fig. 2).

### Experimental

Cu(NO_3_)_2_.6H_2_O (0.25 mmol), tetrahydrofuran-2,3,4,5-tetracarboxylic acid (0.25 mmol), and 1,10-pentathroline (0.3 mmol) were dissolved into 30 ml mixed solvent of distilled water and CH_3_OH (1:1). The solution was heated to reflux for 30 min, and then filtered. The filtrate was allowed to evaporate at room temperature. Blue plate crystals of (I) were obtained after three days, the crystals are washed with cold EtOH and dried in the air. A water molecule with the occupation factor of 0.69 is located in the structure. The percentage of H_2_O molecule in the structure has been proved by the 2.5% (Calc. 0.69 H_2_O per unit cell) of weight lost above 123 centigrade degree.

### Refinement

H atoms of water molecule were located in a difference Fourier map and were refined with distance restraints [O—H = 0.85 (2) and H···H = 1.48 (2) Å]. The occupancy of the water molecule was fixed to 0.69 according to the TG result. Other H atoms were positioned geometrically (C—H = 0.93–0.98 and O—H = 0.82 Å) and refined using a riding model, with *U*_iso_(H) = 1.2*U*_eq_(C, O).

### Crystal data

**Table:**

[Cu(C_8_H_6_O_9_)(C_12_H_8_N_2_)]·0.69H_2_O	*F*(000) = 1023.6
*M**_r_* = 502.30	*D*_x_ = 1.717 Mg m^−^^3^
Monoclinic, *P*2_1_/*n*	Mo *K*α radiation, λ = 0.71073 Å
Hall symbol: -P 2yn	Cell parameters from 6566 reflections
*a* = 12.9215 (7) Å	θ = 2.6–27.5°
*b* = 8.5454 (5) Å	µ = 1.19 mm^−^^1^
*c* = 17.597 (1) Å	*T* = 296 K
β = 90.960 (3)°	Plate, blue
*V* = 1942.85 (19) Å^3^	0.20 × 0.16 × 0.08 mm
*Z* = 4	

### Data collection

**Table:**

Bruker SMART CCD area-detector diffractometer	4444 independent reflections
Radiation source: fine-focus sealed tube	3829 reflections with *I* > 2σ(*I*)
graphite	*R*_int_ = 0.020
φ and ω scans	θ_max_ = 27.5°, θ_min_ = 1.9°
Absorption correction: multi-scan (SADABS; Sheldrick, 1996)	*h* = −16→16
*T*_min_ = 0.797, *T*_max_ = 0.910	*k* = −11→11
24533 measured reflections	*l* = −22→20

### Refinement

**Table:**

Refinement on *F*^2^	Primary atom site location: structure-invariant direct methods
Least-squares matrix: full	Secondary atom site location: difference Fourier map
*R*[*F*^2^ > 2σ(*F*^2^)] = 0.034	Hydrogen site location: inferred from neighbouring sites
*wR*(*F*^2^) = 0.104	H atoms treated by a mixture of independent and constrained refinement
*S* = 1.06	*w* = 1/[σ^2^(*F*_o_^2^) + (0.0625*P*)^2^ + 0.9393*P*] where *P* = (*F*_o_^2^ + 2*F*_c_^2^)/3
4444 reflections	(Δ/σ)_max_ = 0.001
306 parameters	Δρ_max_ = 0.91 e Å^−^^3^
3 restraints	Δρ_min_ = −0.39 e Å^−^^3^

### Special details

**Table:**

Geometry. All e.s.d.'s (except the e.s.d. in the dihedral angle between two l.s. planes) are estimated using the full covariance matrix. The cell e.s.d.'s are taken into account individually in the estimation of e.s.d.'s in distances, angles and torsion angles; correlations between e.s.d.'s in cell parameters are only used when they are defined by crystal symmetry. An approximate (isotropic) treatment of cell e.s.d.'s is used for estimating e.s.d.'s involving l.s. planes.
Refinement. Refinement of *F*^2^ against ALL reflections. The weighted *R*-factor *wR* and goodness of fit *S* are based on *F*^2^, conventional *R*-factors *R* are based on *F*, with *F* set to zero for negative *F*^2^. The threshold expression of *F*^2^ > σ(*F*^2^) is used only for calculating *R*-factors(gt) *etc*. and is not relevant to the choice of reflections for refinement. *R*-factors based on *F*^2^ are statistically about twice as large as those based on *F*, and *R*- factors based on ALL data will be even larger.

### Fractional atomic coordinates and isotropic or equivalent isotropic displacement parameters (Å^2^)

**Table:**

	*x*	*y*	*z*	*U*_iso_*/*U*_eq_	Occ. (<1)
Cu1	0.871218 (18)	0.24503 (2)	0.184653 (12)	0.02868 (10)	
C1	1.01853 (16)	0.2951 (2)	0.30370 (12)	0.0349 (4)	
C2	1.03192 (14)	0.1182 (2)	0.30175 (10)	0.0278 (4)	
H2	1.1041	0.0927	0.2907	0.033*	
C3	1.00314 (14)	0.0481 (2)	0.37973 (10)	0.0277 (4)	
H3A	0.9546	0.1204	0.4036	0.033*	
C4	1.09486 (16)	0.0275 (2)	0.43410 (11)	0.0345 (4)	
C5	0.94334 (15)	−0.1007 (2)	0.35956 (11)	0.0295 (4)	
H5	0.8924	−0.1258	0.3984	0.035*	
C6	1.01493 (19)	−0.2386 (2)	0.34529 (14)	0.0381 (5)	
C7	0.89004 (14)	−0.0507 (2)	0.28438 (10)	0.0270 (4)	
H7	0.8764	−0.1442	0.2535	0.032*	
C8	0.78735 (14)	0.0374 (2)	0.29592 (10)	0.0272 (4)	
O1	0.95434 (13)	0.36239 (16)	0.26139 (9)	0.0423 (4)	
O2	1.07474 (14)	0.36491 (17)	0.35212 (10)	0.0516 (4)	
O3	1.15462 (14)	0.15169 (19)	0.44451 (10)	0.0527 (4)	
H3	1.1322	0.2247	0.4189	0.063*	
O4	1.11204 (13)	−0.09137 (19)	0.46799 (9)	0.0458 (4)	
O5	1.0856 (2)	−0.2330 (2)	0.30242 (14)	0.0725 (7)	
O6	0.98836 (14)	−0.36266 (18)	0.38404 (11)	0.0508 (4)	
H6	1.0209	−0.4387	0.3690	0.061*	
O7	0.77478 (11)	0.16687 (17)	0.26127 (8)	0.0361 (3)	
O8	0.72265 (10)	−0.02515 (17)	0.33703 (8)	0.0343 (3)	
O9	0.96504 (10)	0.04507 (15)	0.24653 (7)	0.0271 (3)	
N1	0.80817 (13)	0.1145 (2)	0.09986 (9)	0.0353 (4)	
N2	0.97214 (14)	0.2981 (2)	0.10238 (11)	0.0397 (4)	
C9	0.72683 (18)	0.0188 (3)	0.10127 (15)	0.0476 (5)	
H9	0.6918	0.0066	0.1466	0.057*	
C10	0.6920 (2)	−0.0641 (3)	0.03716 (19)	0.0641 (8)	
H10	0.6352	−0.1305	0.0405	0.077*	
C11	0.7409 (3)	−0.0473 (3)	−0.02927 (18)	0.0666 (8)	
H11	0.7177	−0.1016	−0.0721	0.080*	
C12	0.8263 (2)	0.0516 (3)	−0.03391 (13)	0.0585 (8)	
C13	0.8850 (3)	0.0778 (4)	−0.10042 (16)	0.0767 (10)	
H13	0.8647	0.0298	−0.1457	0.092*	
C14	0.9682 (3)	0.1693 (4)	−0.09929 (15)	0.0776 (12)	
H14	1.0041	0.1835	−0.1441	0.093*	
C15	1.0052 (3)	0.2479 (3)	−0.03111 (18)	0.0621 (9)	
C16	1.0919 (3)	0.3406 (4)	−0.0257 (2)	0.0772 (11)	
H16	1.1322	0.3564	−0.0684	0.093*	
C17	1.1194 (2)	0.4099 (4)	0.0416 (2)	0.0764 (11)	
H17	1.1789	0.4709	0.0452	0.092*	
C18	1.05587 (19)	0.3877 (3)	0.10668 (18)	0.0587 (7)	
H18	1.0735	0.4364	0.1523	0.070*	
C19	0.9471 (2)	0.2285 (3)	0.03524 (13)	0.0436 (6)	
C20	0.85840 (18)	0.1310 (3)	0.03349 (11)	0.0406 (5)	
O10	0.2766 (4)	0.0628 (9)	0.2674 (5)	0.152 (2)	0.69
H10A	0.254 (7)	0.154 (5)	0.250 (6)	0.183*	0.69
H10B	0.252 (7)	−0.029 (4)	0.265 (6)	0.183*	0.69

### Atomic displacement parameters (Å^2^)

**Table:**

	*U*^11^	*U*^22^	*U*^33^	*U*^12^	*U*^13^	*U*^23^
Cu1	0.03234 (15)	0.02857 (15)	0.02512 (15)	−0.00120 (8)	0.00022 (10)	0.00266 (8)
C1	0.0418 (11)	0.0240 (9)	0.0384 (10)	−0.0040 (8)	−0.0064 (9)	0.0026 (8)
C2	0.0296 (8)	0.0238 (8)	0.0300 (9)	−0.0004 (7)	−0.0021 (7)	−0.0006 (7)
C3	0.0336 (9)	0.0226 (8)	0.0270 (8)	0.0035 (7)	−0.0007 (7)	−0.0013 (7)
C4	0.0380 (10)	0.0323 (10)	0.0332 (10)	0.0039 (8)	−0.0037 (8)	0.0007 (8)
C5	0.0346 (9)	0.0239 (8)	0.0301 (9)	0.0016 (7)	0.0031 (7)	0.0020 (7)
C6	0.0474 (12)	0.0248 (10)	0.0420 (12)	0.0042 (8)	−0.0035 (10)	0.0000 (8)
C7	0.0331 (9)	0.0213 (8)	0.0267 (8)	−0.0034 (7)	0.0035 (7)	−0.0029 (6)
C8	0.0315 (9)	0.0275 (9)	0.0225 (8)	−0.0021 (7)	−0.0008 (7)	−0.0041 (7)
O1	0.0551 (9)	0.0250 (7)	0.0461 (8)	−0.0004 (6)	−0.0193 (7)	0.0028 (6)
O2	0.0656 (11)	0.0234 (7)	0.0646 (10)	−0.0047 (7)	−0.0331 (9)	0.0008 (7)
O3	0.0572 (10)	0.0370 (8)	0.0629 (10)	−0.0039 (7)	−0.0292 (8)	0.0070 (7)
O4	0.0514 (9)	0.0397 (8)	0.0459 (9)	0.0053 (7)	−0.0104 (7)	0.0108 (7)
O5	0.0888 (16)	0.0467 (11)	0.0834 (15)	0.0316 (10)	0.0424 (13)	0.0149 (9)
O6	0.0561 (10)	0.0223 (7)	0.0738 (12)	0.0018 (7)	−0.0037 (8)	0.0087 (7)
O7	0.0374 (7)	0.0350 (7)	0.0361 (7)	0.0080 (6)	0.0074 (6)	0.0078 (6)
O8	0.0347 (7)	0.0350 (7)	0.0334 (7)	−0.0061 (6)	0.0072 (6)	−0.0018 (6)
O9	0.0315 (6)	0.0257 (6)	0.0242 (6)	−0.0027 (5)	0.0033 (5)	−0.0019 (5)
N1	0.0404 (9)	0.0342 (9)	0.0311 (8)	0.0079 (7)	−0.0029 (7)	−0.0030 (7)
N2	0.0337 (9)	0.0369 (9)	0.0488 (10)	0.0056 (7)	0.0069 (8)	0.0160 (8)
C9	0.0450 (12)	0.0412 (12)	0.0563 (14)	0.0001 (10)	−0.0087 (10)	−0.0069 (11)
C10	0.0611 (16)	0.0495 (15)	0.081 (2)	0.0049 (12)	−0.0246 (15)	−0.0201 (14)
C11	0.081 (2)	0.0538 (16)	0.0642 (18)	0.0209 (15)	−0.0334 (16)	−0.0241 (14)
C12	0.0860 (19)	0.0555 (15)	0.0337 (11)	0.0405 (15)	−0.0073 (12)	−0.0057 (10)
C13	0.117 (3)	0.077 (2)	0.0367 (14)	0.039 (2)	0.0063 (16)	−0.0066 (14)
C14	0.122 (3)	0.077 (2)	0.0342 (13)	0.054 (2)	0.0328 (16)	0.0123 (14)
C15	0.0724 (19)	0.0581 (17)	0.0568 (16)	0.0342 (14)	0.0349 (15)	0.0264 (12)
C16	0.074 (2)	0.074 (2)	0.084 (2)	0.0359 (18)	0.0415 (18)	0.0384 (19)
C17	0.0376 (13)	0.0631 (18)	0.129 (3)	0.0090 (12)	0.0241 (16)	0.050 (2)
C18	0.0396 (12)	0.0516 (14)	0.0852 (19)	0.0005 (11)	0.0057 (12)	0.0284 (14)
C19	0.0554 (14)	0.0421 (12)	0.0337 (11)	0.0246 (10)	0.0138 (10)	0.0129 (9)
C20	0.0537 (12)	0.0383 (11)	0.0299 (10)	0.0203 (10)	−0.0003 (9)	0.0014 (8)
O10	0.075 (3)	0.173 (6)	0.211 (6)	0.005 (4)	0.054 (3)	0.054 (6)

### Geometric parameters (Å, °)

**Table:**

Cu1—O7	1.9680 (14)	O6—H6	0.8200
Cu1—O1	1.9835 (15)	O8—Cu1^ii^	2.3363 (14)
Cu1—N2	2.0164 (18)	N1—C9	1.332 (3)
Cu1—N1	2.0235 (17)	N1—C20	1.353 (3)
Cu1—O8^i^	2.3363 (14)	N2—C18	1.326 (3)
Cu1—O9	2.3519 (13)	N2—C19	1.357 (3)
C1—O1	1.246 (2)	C9—C10	1.400 (4)
C1—O2	1.261 (3)	C9—H9	0.9300
C1—C2	1.522 (3)	C10—C11	1.346 (5)
C2—O9	1.433 (2)	C10—H10	0.9300
C2—C3	1.548 (2)	C11—C12	1.394 (5)
C2—H2	0.9800	C11—H11	0.9300
C3—C4	1.521 (3)	C12—C20	1.422 (3)
C3—C5	1.527 (3)	C12—C13	1.423 (4)
C3—H3A	0.9800	C13—C14	1.329 (5)
C4—O4	1.197 (3)	C13—H13	0.9300
C4—O3	1.323 (3)	C14—C15	1.449 (5)
C5—C6	1.521 (3)	C14—H14	0.9300
C5—C7	1.542 (3)	C15—C16	1.374 (5)
C5—H5	0.9800	C15—C19	1.408 (3)
C6—O5	1.195 (3)	C16—C17	1.367 (5)
C6—O6	1.309 (3)	C16—H16	0.9300
C7—O9	1.440 (2)	C17—C18	1.433 (4)
C7—C8	1.542 (3)	C17—H17	0.9300
C7—H7	0.9800	C18—H18	0.9300
C8—O8	1.236 (2)	C19—C20	1.417 (4)
C8—O7	1.272 (2)	O10—H10A	0.89 (2)
O3—H3	0.8200	O10—H10B	0.85 (2)
			
O7—Cu1—O1	92.69 (7)	C1—O1—Cu1	121.38 (13)
O7—Cu1—N2	173.13 (7)	C4—O3—H3	109.5
O1—Cu1—N2	91.47 (8)	C6—O6—H6	109.5
O7—Cu1—N1	93.81 (7)	C8—O7—Cu1	123.11 (12)
O1—Cu1—N1	170.96 (7)	C8—O8—Cu1^ii^	128.49 (12)
N2—Cu1—N1	81.48 (8)	C2—O9—C7	109.69 (13)
O7—Cu1—O8^i^	93.68 (5)	C2—O9—Cu1	107.31 (10)
O1—Cu1—O8^i^	87.70 (6)	C7—O9—Cu1	106.35 (10)
N2—Cu1—O8^i^	91.94 (6)	C9—N1—C20	118.0 (2)
N1—Cu1—O8^i^	98.13 (6)	C9—N1—Cu1	129.25 (16)
O7—Cu1—O9	76.32 (5)	C20—N1—Cu1	112.71 (15)
O1—Cu1—O9	77.47 (5)	C18—N2—C19	119.0 (2)
N2—Cu1—O9	99.28 (6)	C18—N2—Cu1	128.7 (2)
N1—Cu1—O9	97.98 (6)	C19—N2—Cu1	112.27 (16)
O8^i^—Cu1—O9	161.54 (5)	N1—C9—C10	122.6 (3)
O1—C1—O2	123.73 (19)	N1—C9—H9	118.7
O1—C1—C2	121.33 (18)	C10—C9—H9	118.7
O2—C1—C2	114.90 (17)	C11—C10—C9	119.8 (3)
O9—C2—C1	112.38 (15)	C11—C10—H10	120.1
O9—C2—C3	106.34 (14)	C9—C10—H10	120.1
C1—C2—C3	109.59 (15)	C10—C11—C12	120.0 (2)
O9—C2—H2	109.5	C10—C11—H11	120.0
C1—C2—H2	109.5	C12—C11—H11	120.0
C3—C2—H2	109.5	C11—C12—C20	117.4 (3)
C4—C3—C5	115.85 (15)	C11—C12—C13	125.3 (3)
C4—C3—C2	113.96 (16)	C20—C12—C13	117.3 (3)
C5—C3—C2	104.14 (14)	C14—C13—C12	121.5 (3)
C4—C3—H3A	107.5	C14—C13—H13	119.3
C5—C3—H3A	107.5	C12—C13—H13	119.3
C2—C3—H3A	107.5	C13—C14—C15	122.7 (3)
O4—C4—O3	120.58 (19)	C13—C14—H14	118.6
O4—C4—C3	123.20 (19)	C15—C14—H14	118.6
O3—C4—C3	116.18 (17)	C16—C15—C19	117.1 (3)
C6—C5—C3	112.12 (16)	C16—C15—C14	125.6 (3)
C6—C5—C7	109.70 (16)	C19—C15—C14	117.3 (3)
C3—C5—C7	100.71 (14)	C17—C16—C15	120.6 (3)
C6—C5—H5	111.3	C17—C16—H16	119.7
C3—C5—H5	111.3	C15—C16—H16	119.7
C7—C5—H5	111.3	C16—C17—C18	119.4 (3)
O5—C6—O6	124.8 (2)	C16—C17—H17	120.3
O5—C6—C5	123.10 (19)	C18—C17—H17	120.3
O6—C6—C5	112.1 (2)	N2—C18—C17	120.6 (3)
O9—C7—C5	105.07 (14)	N2—C18—H18	119.7
O9—C7—C8	111.73 (14)	C17—C18—H18	119.7
C5—C7—C8	113.30 (14)	N2—C19—C15	123.2 (3)
O9—C7—H7	108.9	N2—C19—C20	117.23 (19)
C5—C7—H7	108.9	C15—C19—C20	119.6 (3)
C8—C7—H7	108.9	N1—C20—C19	116.2 (2)
O8—C8—O7	125.07 (18)	N1—C20—C12	122.2 (2)
O8—C8—C7	117.21 (16)	C19—C20—C12	121.5 (2)
O7—C8—C7	117.70 (15)	H10A—O10—H10B	133 (6)
			
O1—C1—C2—O9	0.9 (3)	N1—Cu1—O9—C7	−73.04 (11)
O2—C1—C2—O9	179.11 (18)	O8^i^—Cu1—O9—C7	77.61 (18)
O1—C1—C2—C3	−117.1 (2)	O7—Cu1—N1—C9	2.64 (19)
O2—C1—C2—C3	61.1 (2)	O1—Cu1—N1—C9	138.5 (4)
O9—C2—C3—C4	144.82 (15)	N2—Cu1—N1—C9	177.6 (2)
C1—C2—C3—C4	−93.47 (19)	O8^i^—Cu1—N1—C9	−91.63 (19)
O9—C2—C3—C5	17.66 (18)	O9—Cu1—N1—C9	79.35 (19)
C1—C2—C3—C5	139.37 (16)	O7—Cu1—N1—C20	−177.48 (14)
C5—C3—C4—O4	−10.3 (3)	O1—Cu1—N1—C20	−41.6 (5)
C2—C3—C4—O4	−131.1 (2)	N2—Cu1—N1—C20	−2.51 (14)
C5—C3—C4—O3	171.93 (18)	O8^i^—Cu1—N1—C20	88.24 (14)
C2—C3—C4—O3	51.1 (2)	O9—Cu1—N1—C20	−100.77 (14)
C4—C3—C5—C6	−42.0 (2)	O7—Cu1—N2—C18	−130.7 (5)
C2—C3—C5—C6	83.97 (19)	O1—Cu1—N2—C18	−3.3 (2)
C4—C3—C5—C7	−158.57 (16)	N1—Cu1—N2—C18	−177.7 (2)
C2—C3—C5—C7	−32.60 (17)	O8^i^—Cu1—N2—C18	84.4 (2)
C3—C5—C6—O5	−51.7 (3)	O9—Cu1—N2—C18	−80.9 (2)
C7—C5—C6—O5	59.3 (3)	O7—Cu1—N2—C19	48.8 (6)
C3—C5—C6—O6	129.65 (19)	O1—Cu1—N2—C19	176.18 (14)
C7—C5—C6—O6	−119.3 (2)	N1—Cu1—N2—C19	1.86 (14)
C6—C5—C7—O9	−81.17 (18)	O8^i^—Cu1—N2—C19	−96.07 (14)
C3—C5—C7—O9	37.17 (17)	O9—Cu1—N2—C19	98.63 (14)
C6—C5—C7—C8	156.60 (16)	C20—N1—C9—C10	−0.1 (3)
C3—C5—C7—C8	−85.06 (17)	Cu1—N1—C9—C10	179.79 (18)
O9—C7—C8—O8	−170.15 (15)	N1—C9—C10—C11	−0.5 (4)
C5—C7—C8—O8	−51.7 (2)	C9—C10—C11—C12	0.4 (4)
O9—C7—C8—O7	11.3 (2)	C10—C11—C12—C20	0.3 (4)
C5—C7—C8—O7	129.76 (17)	C10—C11—C12—C13	179.3 (3)
O2—C1—O1—Cu1	178.38 (18)	C11—C12—C13—C14	−177.2 (3)
C2—C1—O1—Cu1	−3.6 (3)	C20—C12—C13—C14	1.8 (4)
O7—Cu1—O1—C1	78.65 (18)	C12—C13—C14—C15	0.2 (5)
N2—Cu1—O1—C1	−95.88 (18)	C13—C14—C15—C16	178.3 (3)
N1—Cu1—O1—C1	−57.3 (5)	C13—C14—C15—C19	−2.2 (4)
O8^i^—Cu1—O1—C1	172.23 (18)	C19—C15—C16—C17	0.1 (4)
O9—Cu1—O1—C1	3.30 (17)	C14—C15—C16—C17	179.7 (3)
O8—C8—O7—Cu1	−170.39 (14)	C15—C16—C17—C18	−1.2 (4)
C7—C8—O7—Cu1	8.0 (2)	C19—N2—C18—C17	−0.4 (3)
O1—Cu1—O7—C8	−91.67 (15)	Cu1—N2—C18—C17	179.09 (18)
N2—Cu1—O7—C8	35.6 (6)	C16—C17—C18—N2	1.4 (4)
N1—Cu1—O7—C8	82.04 (15)	C18—N2—C19—C15	−0.8 (3)
O8^i^—Cu1—O7—C8	−179.54 (15)	Cu1—N2—C19—C15	179.66 (17)
O9—Cu1—O7—C8	−15.26 (14)	C18—N2—C19—C20	178.61 (19)
O7—C8—O8—Cu1^ii^	111.36 (19)	Cu1—N2—C19—C20	−1.0 (2)
C7—C8—O8—Cu1^ii^	−67.1 (2)	C16—C15—C19—N2	0.9 (3)
C1—C2—O9—C7	−113.58 (17)	C14—C15—C19—N2	−178.7 (2)
C3—C2—O9—C7	6.33 (18)	C16—C15—C19—C20	−178.5 (2)
C1—C2—O9—Cu1	1.55 (17)	C14—C15—C19—C20	2.0 (3)
C3—C2—O9—Cu1	121.46 (11)	C9—N1—C20—C19	−177.37 (19)
C5—C7—O9—C2	−27.77 (17)	Cu1—N1—C20—C19	2.7 (2)
C8—C7—O9—C2	95.47 (16)	C9—N1—C20—C12	0.8 (3)
C5—C7—O9—Cu1	−143.52 (11)	Cu1—N1—C20—C12	−179.11 (16)
C8—C7—O9—Cu1	−20.27 (15)	N2—C19—C20—N1	−1.2 (3)
O7—Cu1—O9—C2	−98.38 (11)	C15—C19—C20—N1	178.19 (19)
O1—Cu1—O9—C2	−2.43 (11)	N2—C19—C20—C12	−179.37 (19)
N2—Cu1—O9—C2	87.02 (12)	C15—C19—C20—C12	0.0 (3)
N1—Cu1—O9—C2	169.62 (11)	C11—C12—C20—N1	−0.9 (3)
O8^i^—Cu1—O9—C2	−39.7 (2)	C13—C12—C20—N1	180.0 (2)
O7—Cu1—O9—C7	18.96 (10)	C11—C12—C20—C19	177.2 (2)
O1—Cu1—O9—C7	114.90 (11)	C13—C12—C20—C19	−2.0 (3)
N2—Cu1—O9—C7	−155.65 (11)		

Symmetry codes: (i) −*x*+3/2, *y*+1/2, −*z*+1/2; (ii) −*x*+3/2, *y*−1/2, −*z*+1/2.

### Hydrogen-bond geometry (Å, °)

**Table:**

*D*—H···*A*	*D*—H	H···*A*	*D*···*A*	*D*—H···*A*
O10—H10A···O5^i^	0.89 (2)	2.47 (10)	2.793 (6)	102 (7)
O6—H6···O2^iii^	0.82	1.84	2.646 (2)	166.
O3—H3···O2	0.82	1.83	2.641 (2)	172.

Symmetry codes: (i) −*x*+3/2, *y*+1/2, −*z*+1/2; (iii) *x*, *y*−1, *z*.
